# Supplementary material for: Cytotoxic CD8+ T Cells Are Involved in the Thrombo-Inflammatory Response during First-Diagnosed Atrial Fibrillation
Source: Cells. 2022 Dec 29;12(1):141. doi: 10.3390/cells12010141 (PMC9818535; doi:10.3390/cells12010141)
Supplement: Supplementary file 1 [file cells-12-00141-s001.zip › cells-2089610-supplementary Figure S1.pdf]

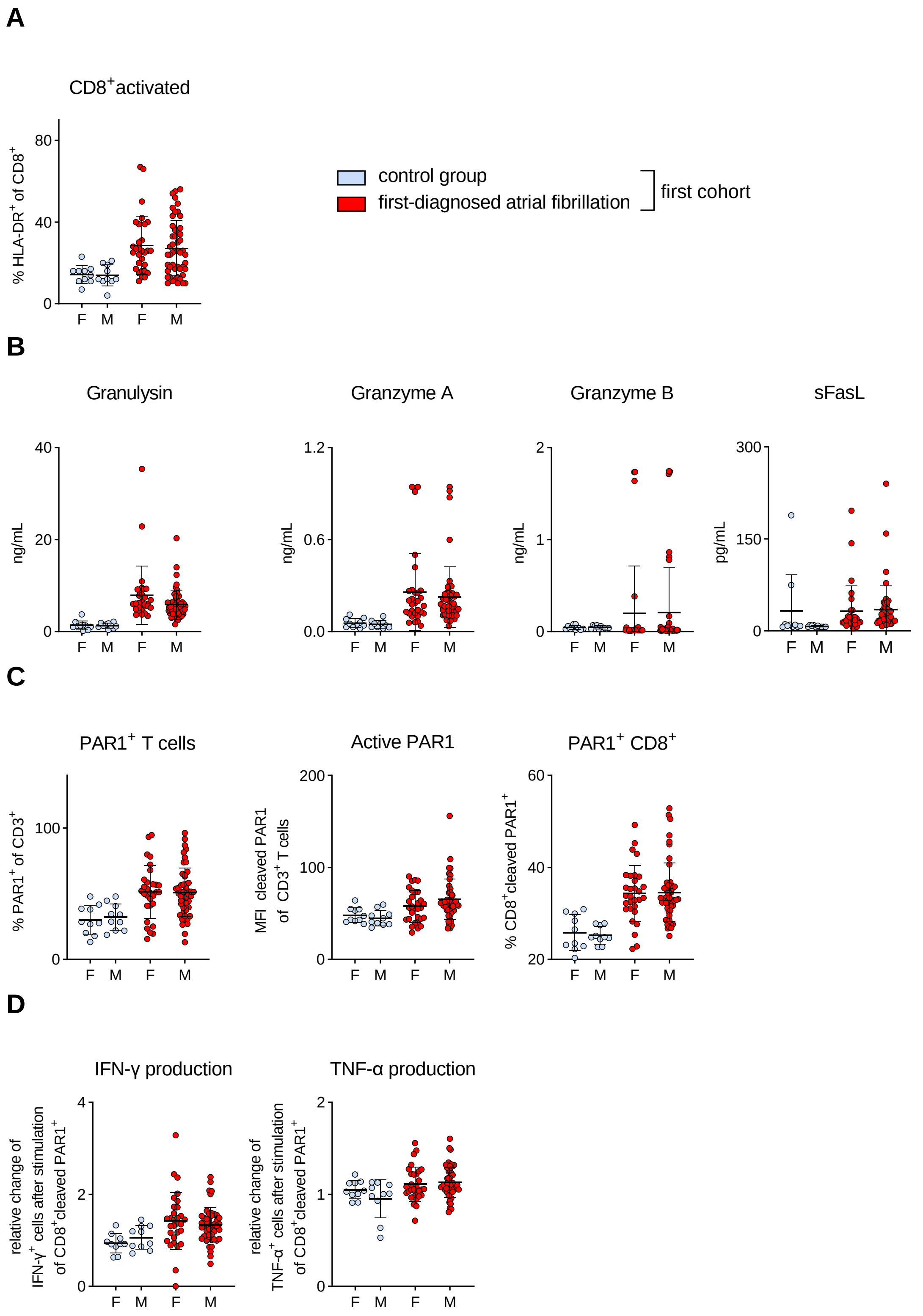

Figure S1. Consideration of gender-specific aspects. F, female; M, male. Patients with first-diagnosed AF vs. control group. (**A,B**) Activation of CD8+ T cells in patients with first-diagnosed AF. (**A**) Phenotyping of circulating CD8+ T cells (flow cytometry). Activated cytotoxic T cells (HLA-DR+). (**B**) Increased plasma levels of cytotoxic effector molecules associated with CD8+ T cell function (ELISA). (**C,D**) CD8+ T cell-mediated effector function in early AF is linked to PAR1 activation. (**C**) Phenotyping via flow cytometry of T cells in patients with first-diagnosed AF revealed a higher percentage of circulating cells that possess PAR1 (CD3+PAR1+), an increased expression of thrombin-activated PAR1 (as expressed by increased mean fluorescence intensity (MFI) of thrombin-cleaved PAR1) and a higher percentage of cytotoxic T cells that express PAR1, which was activated by thrombin (CD8+ cleaved

PAR1$^+$). (**D**) Enhanced pro-inflammatory effector function of thrombin-activated CD8$^+$PAR1$^+$ T cells as expressed by staining for IFN-γ and TNF-α. Results are expressed as single values (n = 10/10/30/50), mean with SD.
